# The theory of expanded, extended, and enhanced opportunities for youth physical activity promotion

**DOI:** 10.1186/s12966-016-0442-2

**Published:** 2016-11-16

**Authors:** Michael W. Beets, Anthony Okely, R. Glenn Weaver, Collin Webster, David Lubans, Tim Brusseau, Russ Carson, Dylan P. Cliff

**Affiliations:** 1 Arnold School of Public Health, University of South Carolina, 921 Assembly St, 1st Flr Suite, RM 131, Columbia, SC 29208 USA; 2 Early Start Research Institute, Faculty of Social Sciences, University of Wollongong, Wollongong, NSW Australia; 3 Department of Physical Education, University of South Carolina, Columbia, SC USA; 4 Priority Research Centre for Physical Activity and Nutrition, Faculty of Education and Arts, University of Newcastle, Newcastle, Australia; 5 Physical Activity Research Laboratory, Department of Health, Kinesiology, and Recreation, University of Utah, Salt Lake City, UT USA; 6 University of Northern Colorado, Greeley, CO USA; 7 Illawarra Health and Medical Research Institute, University of Wollongong, Wollongong, NSW Australia

**Keywords:** Adolescents, Children, Intervention, Moderate-to-vigorous, Obesity, Programs, Schools, Sedentary

## Abstract

**Background:**

Physical activity interventions targeting children and adolescents (≤18 years) often focus on complex intra- and inter-personal behavioral constructs, social-ecological frameworks, or some combination of both. Recently published meta-analytical reviews and large-scale randomized controlled trials have demonstrated that these intervention approaches have largely produced minimal or no improvements in young people’s physical activity levels.

**Discussion:**

In this paper, we propose that the main reason for previous studies’ limited effects is that fundamental mechanisms that lead to change in youth physical activity have often been overlooked or misunderstood. Evidence from observational and experimental studies is presented to support the development of a new theory positing that the primary mechanisms of change in many youth physical activity interventions are approaches that fall into one of the following three categories: (a) the *expansion* of opportunities for youth to be active by the inclusion of a new occasion to be active, (b) the *extension* of an existing physical activity opportunity by increasing the amount of time allocated for that opportunity, and/or (c) the *enhancement* of existing physical activity opportunities through strategies designed to increase physical activity above routine practice. Their application and considerations for intervention design and interpretation are presented.

**Summary:**

The utility of these mechanisms, referred to as the Theory of Expanded, Extended, and Enhanced Opportunities (TEO), is demonstrated in their parsimony, logical appeal, support with empirical evidence, and the direct and immediate application to numerous settings and contexts. The TEO offers a new way to understand youth physical activity behaviors and provides a common taxonomy by which interventionists can identify appropriate targets for interventions across different settings and contexts. We believe the formalization of the TEO concepts will propel them to the forefront in the design of future intervention studies and through their use, lead to a greater impact on youth activity behaviors than what has been demonstrated in previous studies.

## Background

Over the past four decades, numerous physical activity interventions targeting young people (≤18 years) have been developed, implemented, evaluated, and, to a lesser extent disseminated [1–4]. Best practice for intervention development is the a priori use of behavioral theory to target or use theoretically relevant constructs or approaches that, when modified through exposure to the intervention, result in increased levels of youth physical activity [5]. Historically, behavioral theories have largely focused on complex intra- (e.g., autonomy, self-efficacy) and inter- (e.g., peer social support) personal processes mediating youth physical activity behaviors [6–10]. More recently, the introduction of social-ecological models has expanded the targets of interventions to multiple levels of the environments and settings where youth spend a majority of their time [11–13].

While interventions guided by such theories or models have helped in the identification of challenges associated with increasing youth physical activity, these interventions have rarely demonstrated changes in the mediators targeted [6–8] and generally have had little influence on youth physical activity behavior [14, 15]. A recent review indicates that youth physical activity interventions have produced a small effect of approximately 4 more minutes of moderate-to-vigorous physical activity (MVPA) per day [16]. Further, some of the largest and most recently conducted interventions founded on complex intra- or inter-personal behavioral theories [17, 18] or ecological models [15, 19–22] have resulted in limited changes in youth physical activity or sedentary behaviors. Thus, the effectiveness of existing theory-based intervention approaches has been marginal and, in some instances, such results were not altogether unexpected by those who designed the intervention [23].

Along with the use of complex behavioral theories and ecological models to evoke behavior change, youth physical activity interventions have often employed additional approaches with salient features that appear more pragmatic than theoretical. Such approaches typically involve expanding physical activity opportunities (e.g., adding new physical activity opportunities before or after school), extending physical activity opportunities (e.g., adding additional time for existing physical activity opportunities, such as elongating recess), and/or enhancing physical activity opportunities (e.g.., augmenting existing physical activity opportunities, such as providing choice within physical education) to maximize the amount of physical activity youth accumulate [24, 25]. While these fit within an overarching ecological framework, too often they lack explicit formalization or are entirely absent from the more traditional theory(ies) or models guiding the development of an intervention. These mechanisms also lack a common taxonomy by which the scientific literature refers to them when applied across various settings and contexts within an intervention. Interventions that do include one or more of these are mostly devoid of explanations about how and why such approaches should lead to changes in youth physical activity [26, 27]. This renders their perceived contribution to the overall effectiveness of an intervention as subsidiary to the classical theoretical constructs or models guiding an intervention’s development.

In this article, we propose that expanding, extending, and enhancing physical activity opportunities for youth are mechanisms that should be foregrounded in the design of future interventions. We argue that these have served as the primary mechanisms of change in many youth physical activity interventions, yet have been given secondary consideration because they lack a formal theoretical framework to meaningfully bind them and demonstrate their complimentary application to physical activity behavior change. Evidence of this oversight can be found in systematic reviews of mechanisms of change in youth physical activity interventions and correlates of youth physical activity, which almost exclusively focus on intra- or inter-personal mediators, even when one or more of these three mechanisms were present within an intervention [8, 28–30]. The reason for this is understandable, given that the selection of theory and its subsequent application becomes the lens by which interventionist use to define the problems and identify solutions to youth physical inactivity [31]. However, we believe that expanding, extending, and enhancing need to be better understood so increased attention can be directed towards them to ensure they are harnessed for maximal impact within intervention design. Moreover, a firmer understanding of how these mechanisms operate can increase their ability to strengthen existing interventions to create high quality physical activity experiences for youth.

It is important to note that the focus on expanding, extending, and enhancing physical activity opportunities does not negate the importance of the traditionally applied behavioral theories and models used in intervention studies to date. In fact, the intra- and inter-personal constructs specified in many behavioral theories are hallmarks of quality, enjoyable physical activity experiences. Thus, we need to continue to identify and maximize the enjoyment of physical activity within any setting. However, these intra- or inter-personal constructs only operate within the context of providing the experience – which is the fundamental theoretical tenet on which the present article is based – youth are more active when there are more opportunities.

The overall aim of this article is to present evidence of the effectiveness of extended, expanded, and enhanced opportunities as it relates to youth physical activity promotion. Examples are drawn from both the observational and experimental literature to provide evidence of their use and effectiveness. We also discuss the practical implications of selecting one or more of these approaches for intervention development. We believe the formalization of these mechanisms into a theoretical framework, which we refer to as the *Theory of Expanded*, *Extended*, *and Enhanced Opportunities* (referred to hereafter as TEO), will heighten youth physical activity interventionists’ understanding of the importance of these as primary drivers of intervention success or failure.

The value-added contribution of formalizing these mechanisms within a theoretical framework is that it provides (a) a unifying language by which the scientific field can refer to approaches that are nested within each of the three mechanisms, (b) a structure for the proposal of a priori hypotheses associated with the mechanisms that can be formally tested within empirical studies, and (c) clear explanations of what mechanisms (i.e., expanded, extended, and enhanced) are logically related to the phenomenon of interest (in this case youth physical activity) and how they are casually related. For the purpose of this article, we draw upon the criteria of what constitutes a theory proposed by Kuhn [32], Dubin [33], Wacker [34, 35], and others [36, 37] to establish the overarching theoretical foundation for the TEO. Broadly, the criteria include operational definitions, domain specificity, set of relationships, and specific predictions [35]. Importantly, theory should be pragmatic, closely linked with practice, parsimonious, and offer an understanding to previously unnoted relationships [31, 33, 35, 38]. Further, any new theory should offer a conceptually distinct set of new relationships which should serve to bridge the gap among existing theories [36]. The following sections present evidence of the utility of the TEO and demonstrate its contribution to the theoretical repertoire behavioral and social scientists can draw from in the design of theory-driven interventions.

## Definitions of expansion, extension, and enhancement

The following operational definitions of expanded, extended and enhanced physical activity opportunities are proposed and used as a guiding framework of the theory and review of the literature. These definitions are based on our understanding of interventionists’ application of the numerous observable approaches that can be classified within the constructs of expand, extend and enhance and, ultimately, how these function within both the observational and experimental literature that spans a number of domains (i.e., settings) which include childcares, schools, before and after school, summer/holiday camps, and sports, where the promotion of youth physical activity is commonly targeted. They are described in detail below and can be found in Table 1.

**Table 1:** Expanded, Extended, and Enhanced definitions and examples

Theoretical Mechanism	Definition	Examples
Expansion	Replacing time allocated for low active or sedentary activities with time allocated for more active activities.	Substituting seatwork with active learning tasks in general education classrooms. Providing a before or after school opportunity to be active, where one did not exist previously.
Extension	Lengthening time currently allocated for physical activity opportunities.	Providing additional physical education (PE) lessons per week, on top of what is currently provided. Lengthening or adding additional recess PE sessions per week or allocating more time for recess or PE on a given day.
Enhancement	Modifying an existing physical activity opportunity to increase the amount of physical activity youth accumulate during an allotted period of time.	Reducing student wait time during PE lessons to increase physical activity Increasing portable equipment options for students during recess. Providing choice among two or more activity opportunities

The first two mechanisms, expansion and extension, represent the replacement of sedentary time with an opportunity for youth to move. The *expansion* of opportunities refers to the introduction of an entirely “new” physical activity opportunity. The new opportunity serves to broaden pre-existing physical activity opportunities, and, correspondingly increase time allotted for youth to be physically active. An important consideration for an approach to be regarded as an expansion is that the introduced opportunity cannot have existed previously within that setting in some form. Examples of expansion from the literature include the introduction of physical activity breaks into a classroom environment, integration of physical activity into other learning areas such as language and mathematics, the introduction of before- or after-school physical activity opportunities, the provision of activity equipment or standing desks within a classroom, the introduction of active learning centers in childcare settings, or having children dropped-off several blocks away from school to facilitate active transport [39–52]. The five-component model for comprehensive school physical activity programs provides a useful guide for the scope of expansion opportunities available [13, 53].

The *extension* of opportunities is defined as allocating additional time for an existing physical activity opportunity. This can be achieved by adding more time to a scheduled opportunity, such as extending a 20 min recess session to 40 min per day or adding additional time for preschoolers to be active indoors, or by adding another opportunity of the same type, such as scheduling physical education (PE) for 3 days per week instead of one day per week or providing two recess breaks per day versus only one [54–59]. It is important to reemphasize the distinction between expansion and extension, as the former is the introduction of a new physical activity opportunity, whereas the latter is the elongation of an existing physical activity opportunity. However, both serve to replace time allotted for sedentary behaviors with alternative, more physically active opportunities.

An *enhancement* of opportunities is defined as the modification of an existing physical activity opportunity to increase the amount of physical activity accumulated during that opportunity. Examples include enhancing the quality of a PE lesson, sport practices, or afterschool or summer/holiday camps to maximize the amount of physical activity that occurs above routine practice or enhancing the playground environment to make it more physically active [57, 60–66]. Enhancements typically involve training supervisors (i.e., teachers, staff, or parents) to modify games or deliver more active lessons, changing curricula to introduce more variety of physical activities for youth, purchasing and making more accessible portable or fixed play equipment, or the use of playground markings to increase youth physical activity.

## Empirical evidence

The following section presents examples drawn from the empirical literature to illustrate the casual relationships among expansion, extension, and enhancement with changes in physical activity within youth physical activity interventions. Evidence is drawn from both observational and experimental studies to support that, either singularly or in combination, expansion, extension, and enhancement have a substantial impact on youth physical activity. A basic assumption (and testable hypothesis) underlying the TEO is that the presence of new activity opportunities (i.e., extension), the elongation of existing activity opportunities (i.e., expansion), and/or the modification of existing activity opportunities to make them more active (i.e., enhancement) will lead to increased physical activity of youth who come into contact with these opportunities. The empirical evidence presented is not intended to serve as an exhaustive review of all published studies applying/investigating one or more of these mechanisms. Rather, examples have been identified to illustrate how these mechanisms theoretically operate in observational research and function to increase youth physical activity in experimental research.

### Expansion

**Fig. 1:**
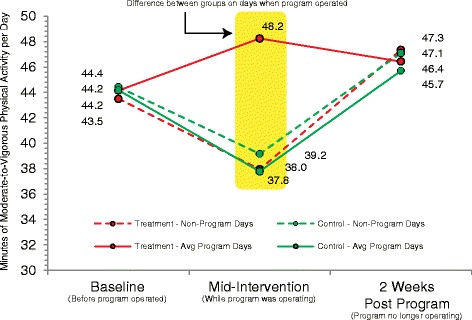
Comparison of minutes of MVPA per day on program and non-program days

Two recent experimental studies provide excellent examples of how expansion operates. These two studies focused on the introduction of a new physical activity opportunity two to three days per week in the hours immediately after the end of the school day [26, 27]. The interventions were grounded in the Self Determination Theory and/or the Social Cognitive Theory and were designed to promote behavioral skills for engaging in physical activity outside of the program, as well as to promote physical activity during the program by offering different choices for physical activity within an autonomy-supported environment. Both studies demonstrated that when the participants attended the program, increases in their physical activity levels were observed. Importantly, both studies reported that once the opportunity ended after the 17- and 20-week trial periods, participants’ activity returned to baseline levels. Further, support of expansion was found when participants’ activity levels on the two or three days that the program was offered were compared to activity levels during non-program days. Both studies observed higher levels of physical activity on days when the program operated over the 17- and 20-week trial compared to days during the week when the program was not offered (see Fig. 1). These studies provide important evidence that the reason for the increase in physical activity was the addition of physical activity opportunities by introducing a new activity opportunity (i.e., expanding) two or three times per week.

Much like adding a new after school program that promotes activity, classroom-based approaches (a form of expansion) increase activity levels by replacing sedentary time in the classroom [67]. These interventions focus on exchanging sedentary activities (i.e., seatwork) with physically active lessons, active transitions, and movement breaks within general education classrooms [41, 68–71]. Not surprisingly, when time allocated for students to be sedentary is replaced with an opportunity to be active, youth are more active – at least within the classroom. A recent quantitative review by Erwin and colleagues [67] on the impact of classroom-based physical activity interventions on youth physical activity found that, compared to routine activity levels in the classroom, classroom-based physical activity interventions elicit an average pooled effect size of 0.99.

### Extension

**Fig. 2:**
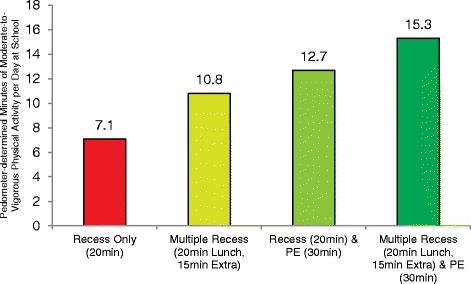
Comparison of minutes of MVPA per day during school across 4 types of physical activity opportunity schedules

For extension, some of the more consistent evidence supporting its utility comes from observational studies comparing youth physical activity levels on school days with and without an existing physical activity opportunity (e.g., PE or recess) [56, 72]. Further, additional studies have identified differing lengths of time allotted for the same opportunity and corresponding physical activity levels of youth [73, 74]. One of the more recent examples of extension comes from a observational study by Brusseau et al. [56]. In this study, the physical activity levels of elementary-age students were compared across school days where various combinations and amounts of existing physical activity opportunities were provided. They found children were most active on days where the most opportunities were available during school, in the form of a 30 min PE class and one or more recesses lasting 15–20 min (12.7 and 15.3 min of MVPA per day), compared to days when only one 20 min recess opportunity was provided (7.1 min of MVPA per day, see Fig. 2). The research literature on segmented school days supports these findings, with children accumulating greater amounts of activity on days where a single PE class or recess session is offered compared to days without these opportunities [72, 75–81].

**Fig. 3:**
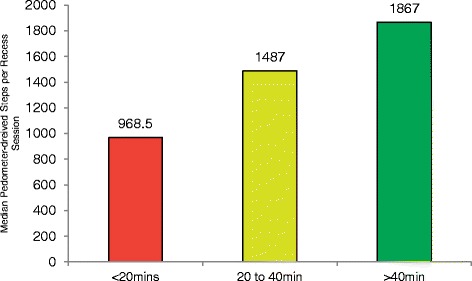
Comparison of steps per recess session across 3 lengths of recess

Fewer studies have compared the activity levels of youth across different lengths of the same physical activity opportunity. One observational study compared the activity levels of students who received three different lengths of recess sessions (see Fig. 3) [73]. This study found that recess durations of 40 min or longer were associated with the greatest amount of physical activity (median 1,867 steps per session), compared to recess durations of 20–40 min (median 1,487 steps per session) and recess lasting less than 20 min (median 968 steps per session) [73]. Other studies support these findings, with children who are provided longer durations of an activity period accumulating a greater amount of activity compared to children who are provided with shorter durations of the activity opportunity [82, 83].

**Fig. 4:**
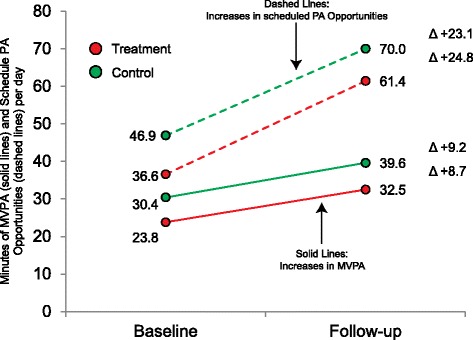
Comparison of minutes of MVPA accumulated and time allocated for physical activity opportunities

There are fewer experimental examples of the impact of the extension approach on youth physical activity. The clearest example was demonstrated in a recently completed group randomized controlled trial in 20 afterschool programs conducted by Craddock et al. [84]. Intervention programs received a multicomponent intervention in addition to increasing the amount of time scheduled for PA opportunities by +24.8 min per day (from 37 to 61 min per day, see Fig. 4). This corresponded with an increase of +8.7 min of MVPA per day (23.8 vs. 32.5 min of MVPA per day) by post-assessment. An important aspect of this study was that the control group, which did not receive any of the intervention, also increased the amount of time scheduled for physical activity opportunities by +23.1 min per day (47–70 min per day) and saw an identical increase in MVPA of +9.2 min per day (30.4–39.6 min of MVPA per day). This study provides key evidence that the extension of the time allotted for pre-existing physical activity opportunities can provide substantial increases in MVPA. This experimental finding is supported by observational studies that show longer physical activity opportunities are associated with higher levels of physical activity among youth [73].

### Enhancement

Modest support of the enhancement approach of the TEO comes from several systematic reviews and meta-analyses of interventions in PE and recess. A review and meta-analysis [61] of interventions in the PE setting found 14 studies that enhanced PE lessons either through curricula adoption, teacher professional development training, or infusing high intensity physical activities into PE lessons. Overall, enhancements led to an average increase of ~10% in the proportion of time spent in MVPA during a PE lesson. For an average PE lesson length of 45 min, capitalizing on enhancement would result in approximately 5 additional minutes of MVPA per lesson [85]. Enhancements in the recess setting show similar promising results. A review [60] of 13 interventions targeting enhancements during recess reported the most common enhancements were adding equipment, painting playground markings or designated play zones, and/or working with teachers to lead or facilitate students’ activity. Of the 13 studies included in the review, 12 reported a statistically significant improvement in students’ activity with the percentage of time spent active during recess increasing from 5% to greater than 30%. Based on these reviews, enhancements can lead to important increases in activity levels within the settings where the enhancements occurred (PE or recess) without an increase in the time allotted for the physical activity opportunity. Further, enhancements have the additional benefits of enhancing teacher professional development, improving quality pedagogy, and enhancing curricula.

### Extension and enhancement

**Fig. 5:**
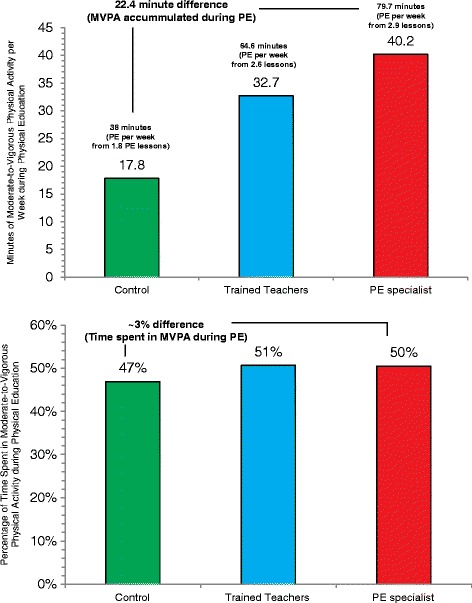
Comparison of weekly minutes of MVPA accumulated during Physical Education and the percentage of time spent in MVPA during Physical Education

In several studies, extension has been incorporated along with another, most commonly enhancement. One of the earliest examples incorporating extension and enhancement comes from the SPARK PE group randomized control trial conducted by Sallis et al. [54, 55]. The intervention focused on providing more PE sessions per week (38 min of PE per week for control schools vs. 65 and 80 min per week for the intervention schools) as well as enhancing the delivery of PE through teacher professional development training focused on maximizing activity during scheduled PE classes. The authors found that children attending schools with the most PE minutes scheduled per week accumulated the greatest amount of MVPA per week (40.2 min per week) compared to schools with the least amount of PE scheduled per week (17.8 min per week, see Fig. 5). The comparison among conditions regarding enhancements was less dramatic, with professional development leading to approximately 50% of class time spent in MVPA, compared to 47% in classes without the enhancement. Had the control schools offered PE at the same rate as the intervention schools (3 days per week), children would have accumulated 37.6 min of MVPA per week (47% of 80 min for PE) compared to the 40 min of MVPA per week accumulated in the intervention schools. It is important to highlight that comparisons of MVPA across conditions were all made at a weekly level. Furthermore, no baseline data were presented in the original study, thus it is difficult to determine the extent of change that occurred as a result of implementing the intervention.

A quasi-experimental study by Moller et al. [86]. also incorporated both extension and enhancement. The intervention extended PE from the standard 90 min per week to 4.5 h per week and targeted enhancing the activity levels obtained by students during PE by using a sport-focused PE curriculum [86]. Estimates of activity showed students attending schools with 4.5 h of PE engaged in 59 and 48.5 min (boys and girls, respectively) of MVPA per week during PE compared to 24.5 and 19 min (boys and girls, respectively) of MVPA per week during PE in those attending schools with the standard 90 min of PE per week. Overall, in-school estimates of activity showed students in schools with 4.5 h of PE per week accumulated 185 and 130 min (boys and girls, respectively) of MVPA per week in-school for students attending the extra PE schools compared to 143 and 100 min (boys and girls, respectively) of MVPA per week for students attending schools with only 90 min PE per week. When examining the impact of enhancement, boys and girls attending intervention schools achieved 21–29% and 17–23% of PE in MVPA, respectively, compared to 26–29% and 20–22% for boys and girls, respectively, in comparison schools. One of the major limitations of this study was no baseline data were collected prior to the increase in PE. Yet, similar to the SPARK study [55], extension was more effective than the enhancement in increasing MVPA. Nevertheless, consistent with previous studies [55, 84], the more time allotted for existing PA opportunities during the week translates into a greater accumulation of activity than the increases observed solely from enhancements.

### Expansion, extension, and enhancement

Finally, an excellent illustration of an intervention that used all three TEO mechanisms can be found in the KISS study (Kinder-Sportstudie) [87]. The intervention involved 3–5 physical activity breaks during academic lessons (expansion), adding two more days of PE per week (3 –5 days, expansion), and augmenting the MVPA elicited during lessons in these two additional PE days by recruiting trained PE specialists to deliver PE as opposed to the regular classroom teacher (enhancement). These changes led to an increase of +7 min of MVPA (38–45 min) per day during school compared to a 5 min decrease in MVPA minutes per day (37–32 min) in the control schools.

## Intervention, practice, and policy considerations when applying the TEO

Although there is considerable evidence supporting that the appropriate use of the TEO leads to increases in youth physical activity, there are a number of considerations that require attention from intervention scientists, practitioners, and policy makers when incorporating them effectively within an intervention to promote youth activity. These considerations are discussed below.

### Target of Intervention

Many youth physical activity interventions focus almost exclusively on intra- or inter-personal behavioral theories targeting the youth participating [17, 18]. Those studies utilizing such theoretical approaches have rarely demonstrated change in the mediating variables associated with subsequent changes in youth physical activity [8]. Hence, we believe these efforts are largely misplaced in assuming children and adolescents are autonomous agents in their physical activity decision making. The literature, however, would suggest that youth are largely active when provided either compulsory activity opportunities or opt to participate in voluntary activity opportunities [26, 27, 56, 62, 67, 72, 77, 88–91]. Because of this, when interventions incorporate one or more of the three approaches, effort should be directed at those involved in making decisions surrounding expansion, extension, or enhancement.

The TEO targets various levels within an ecological model, thereby necessitating different intervention strategies depending on which mechanism is used. For instance, using extension (either to elongate time for a physical activity opportunity or to add additional days during the week that a physical activity opportunity occurs) requires decisions to be made by individuals other than the youth who ultimately participate in and benefit from these opportunities. These decision makers, who could be school administrators or those who oversee program operations, would need to decide whether adding more time for an existing physical activity opportunity would be beneficial, and most importantly, what existing sedentary time this extended opportunity would replace. In terms of school-based interventions, adding more time for PE or recess would be a policy-related decision and come at the cost of reduced time for other, presumably higher priority areas, such as English and Math. Thus, while allocating more time for existing physical activity opportunities like PE and recess have consistent observational and experimental support for increasing youth physical activity, the focus of the intervention should shift from children and adolescents as decision-makers to those who govern the settings or policies the intervention is hoping to alter and how one can get them to extend physical activity opportunities for youth.

Similar considerations need to be made in implementing enhancement and expansion. The majority of enhancements to PE and the addition of classroom-based physical activity interventions have shown increases in youth physical activity. The question then becomes, “What is it about the implementers (the classroom or PE teachers) that needs to be considered for this type of intervention to be implemented and, importantly, sustained over time?” We should also be asking, “What are these implementers willing to do, what skills do they need, and what materials/strategies are they most likely to use on a regular basis? [92]” These are just some of the questions our interventions have yet to adequately address, but are central to maximizing the impact of the expansions or enhancements. Asking classroom teachers to start infusing physical activity into academic lessons or to reserve a period of time for an activity break requires different levels of involvement, commitment, and intervention approaches that largely focus on the teachers and administrators, not the students. An important consideration when designing future studies using expansion or enhancement is whether increased activity should be a primary or process outcome. The literature to date is fairly robust in demonstrating that when more activity opportunities are provided or existing ones enhanced, youth are more active. To augment this literature, it is now paramount to identify how potential implementers conduct their daily work, what such individuals can do to expand and/or enhance physical activity opportunities, and how willing such individuals are to take on implementation responsibilities.

### Voluntary vs. compulsory attendance

Those physical activity opportunities that modify, elongate, or introduce new physical activity opportunities during the school day have the broadest reach since attendance in these opportunities is largely mandatory – they occur when school is in session. The greatest gains from a public health perspective, therefore, will be from the compulsory application of the TEO within the school environment where youth cannot “opt out” of the expanded, extended, or enhanced physical activity opportunities. The literature is fairly robust in regards to increasing physical activity within a setting where the intervention is delivered, yet limited evidence exists on the ability to influence active elsewhere in the day, outside of the setting where the intervention was delivered [93]. Embedding the TEO within the school day may also be most beneficial in helping those youth who are the least motivated to participate in physical activity. For example, a study by Alderman et al. [72] found that the least active middle schoolers (based on the lowest tertile of daily step counts) accumulated 31% more steps on days PE was offered, compared to days without PE. Additional evidence comes from a study by Fairclough et al. [94] who reported that estimates of in-school MVPA were more similar between high and low active 10–11 year-olds (28.4 vs. 23.3 min of MVPA per day, respectively) compared to MVPA accumulated outside of school between high and low active youth (38.4 vs. 29.7 min of MVPA per day, respectively). Thus, the compulsory nature of the activity opportunities, such as PE, recess, and lunch breaks, appears to serve as a necessary factor in getting youth more active, especially those who may not voluntarily participate in an activity experience outside of the structured setting.

When expanding physical activity opportunities outside of the regular school day, attendance at new programs is voluntary and, by its very nature, challenging. Studies that developed new physical activity opportunities in the hours after the school day consistently report attendance is less than optimal [26, 27, 95–97]. However, when youth do attend these voluntary opportunities they exhibit higher levels of physical activity compared to days they do not attend or the program does not operate [26, 27]. Given this, expanding voluntary physical activity opportunities is highly effective at increasing PA for those youth who attend. The question is then, “How do we get more youth to attend (and/or keep attending) such programs?” Extensive formative work went into the development of these voluntary physical activity opportunities to ensure the program provided activities that were either culturally tailored or had other programmatic components that appealed to the target population [26, 95]. Moreover, these studies delivered activities founded in complex behavioral theory designed to maximize motivation for involvement both within and outside of program operating hours [26, 27]. For example, one study [26] conducted extensive formative work to design an after-school program that appealed to girls [98, 99]. However, in the larger-scale trial [26], attendance at the program was minimal and psychosocial outcomes indicated that girls receiving the dance intervention reduced their motivation for physical activity. Collectively across studies, the formative work conducted appeared to minimally influence attendance rates.

Based on this, it seems as a field we are missing something. Perhaps the methods for understanding what youth want out of a physical activity experience are limited. When conducting formative studies, questions are typically focused on understanding the activities the target population enjoys. However, such answers provide little information on whether or not they would be willing to attend a program voluntarily. Further, attendance at voluntary programs may not be a decision within youth’s locus of control. Reasons for not attending may relate more strongly to parental obligations, lack of transportation to and from the opportunity, costs associated with fee-based programs or lack of motivation to participate in such programs. These issues have yet to be answered and are important considerations for those seeking to develop and deliver voluntary physical activity opportunities with substantial reach.

As a field we also have to consider whether developing and operating new programs either before- or after-school is a business in which we should be involved. Operating a program for youth involves more than providing a physical activity experience for a limited period of time. There is liability insurance, enrollment and retention of children, attendance rates, dealing with parent expectations, providing transportation to and from the program, and negotiating with schools for space. These are only a few of the day-to-day operational logistics that have to be continuously monitored to ensure the program runs smoothly. These operational logistics are ones many for- and non-profit organizations, such as YMCAs and Boys and Girls Clubs, have extensive experience with. Further, if a new program is introduced and is financed by outside resources (most likely in the form of a grant), interventionists need to have a firm business plan for the program’s continuation once grant funding ceases. This calls into question the benefit of programs that simply disappear after a few weeks of operation, especially if the improvements in activity levels were entirely due to having access to the program.

### Gains in one setting, losses in another?

**Fig. 6:**
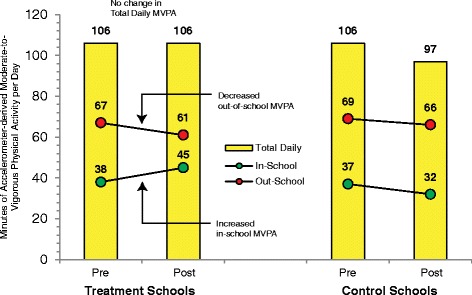
Comparison of minutes of MVPA accumulated in-school, out-of-school, and total daily

An important question that has yet to be fully answered is whether expanding, extending, and/or enhancing PA opportunities in one setting are offset by decreases in activity in another. In the KISS study [87], increases in MVPA were reported during the school day for the intervention schools, yet total daily MVPA was identical from baseline to post-assessment (see Fig. 6). Moller et al. [86] examined 4.5 h per week of PE vs. 90 min per week and showed similar results to the KISS study. In-school estimates of MVPA favored schools with extended PE, but overall daily MVPA levels were identical between groups.

Prior studies suggest youth do not lower their activity levels in one setting when provided with more physical activity opportunities and volume of activity in another [100, 101]. However, other recent studies suggest youth with high amounts of activity on one day compensate by lowering their activity on a subsequent day [102]. Further, some studies suggest there is an activitystat, that hypothesizes children decrease their physical activity in some parts of the day to offset increases in physical activity during other parts of the day, which helps them to maintain a constant level of daily physical activity [101]. It is important to note that most of the studies testing the activitystat hypotheses have been cross-sectional. Although a definitive answer to this is unavailable and further experimental evidence is needed, the evidence from several interventions suggests that targeting increases in physical activity in one setting may not lead to overall improvements in youth activity levels [86, 87].

The potential to compensate for gains in physical activity in one setting by decreasing activity in another is highly problematic for interventionists and future studies need to provide information to inform physical activity promoters as to whether or not such a phenomenon has occurred. While a number of studies provide estimates of total daily physical activity or physical activity by intervention setting (e.g., during the classroom or PE), many do not present both. A consideration for future studies is to report activity outcomes for various time segments during the day when the intervention was delivered, as well as across the entire day [103]. An example of this would be to collect and report classroom physical activity, total in-school physical activity, and total daily physical activity for an intervention focused on classroom physical activity opportunities. Another example would be to provide estimates of physical activity when the program is offered, such as on the 2 or 3 days per week when the program operated, along with the estimates of physical activity when the program was not offered – the remaining 2 or 3 days on non-program days [104]. The utility of presenting activity levels in this way is the ability to examine an intervention’s impact in the targeted setting along with total daily increases or decreases in activity. This would help to avoid erroneous conclusions that interventions were ineffective when in fact the effect may have been suppressed because activity levels were averaged across settings or segments within a day not being targeted by the intervention, or days an intervention did not occur [26, 105, 106]. The collection and reporting of this information would provide evidence on whether an intervention has the desired effect in a given setting or segment of day and whether the intervention contributes to increases in total daily physical activity, which is likely the ultimate goal.

### Which TEO mechanism to use? Practicality, implementation, and cost

It is important to consider that each of the three TEO mechanisms comes with unique considerations regarding practicality, implementation, and cost. For instance, expansion and extension provide additional time for physical activity, yet accomplish this by either adding to pre-existing opportunities or introducing completely new opportunities. Both replace time allotted to other sedentary opportunities, but require different levels/targets for intervention. For instance, adding time to an existing opportunity, such as adding 15 min to an existing PE lesson or recess period, would require a different set of intervention strategies that include working with school or district personnel, compared to training teachers to deliver classroom-based activity breaks or infusing physical activity into an academic curriculum. These differences each have associated practicality considerations, such as whether school officials are willing to replace academic time for additional physical activity time in the form of more recess periods or PE or if classroom teachers are willing to use physical activity breaks or infuse physical activity into academic lessons. Practically, one could argue that it’s more likely classroom teachers would include physical activity in their classrooms rather than school officials altering the time allocated for physical activity by removing time from academics to have more recess or PE, even though essentially both approaches would be removing time from academics, unless activity is infused into the academic routine. In other settings outside of school, such as before- and after-school programs or childcare centers, extending the amount of time allotted for physical activity or expanding physical activity opportunities may be easier to accomplish given the lower external pressures related to academic and learning outcomes.

Implementation and its monitoring would also vary across extension and expansion. For extension, elongating a PE lesson or adding an additional recess each day would, in theory, require less from an implementation approach, since this time segment would be added to a school’s schedule. The key for high implementation would then be to ensure a school “sticks” with its daily schedule. This may require more up-front work with schools and districts to attain buy-in from them to alter a school’s daily schedule. At least one study has shown that schools do not necessarily adhere to their daily schedule for PE, suggesting some need for continuous monitoring at the school level [107]. However, ensuring adherence to the daily schedule may require fewer resources and less complex processes than working with all teachers within a school to utilize some form of physical activity in their classroom at a sufficient frequency to impact youth physical activity [108].

Issues of implementation also relate to monetary costs associated with selecting an approach within one of the three mechanisms. Training classroom teachers to deliver physical activity breaks with fidelity, purchasing equipment or materials, and conducting follow-up visits and trainings may be more costly from an intervention delivery standpoint than working with school or district-level officials to alter school schedules to include additional physical activity opportunities [109]. Thus, while practicality is limited using extension compared to expansion, the overall cost of delivery of extension may be substantially reduced given the targeted level of the intervention as well as a potentially higher level of implementation.

When expansion and extension are not possible, enhancement of existing physical activity opportunities, such as recess and PE, are an option. In terms of practicality, it is high. PE teachers often receive some form of continuing education or professional development training as part of their employment. This time, which is already built into school schedules, could be used towards training teachers to enhance PE. Ensuring high quality implementation of PE enhancements, however, may be difficult and costly. Previous studies have shown that achieving adequate implementation to enhance children’s physical activity may require well trained PE consultants to visit schools once every 2 weeks for 2 years [110] or up to 32 h of professional development training [55]. Further, recent cost analyses show increasing MVPA in PE by 1.9 min for all children in the U.S. would cost approximately $70.7 million during the first year, of which $68 million (96%) would go towards the purchase of curriculum and equipment sets [111]. These costs do not include the extensive hourly training or bi-weekly on-site one-on-one trainings required to achieve a minimal increase in the percentage of time spent in MVPA during PE [55, 110]. Such costs may be outside what schools are willing to pay and little attention has been paid to this issue. Other approaches within enhancement, such as modifying playgrounds with different colored markings or purchasing portable play equipment may require less resources and additional follow-up but the longer-term effectiveness of these approaches is unknown [60].

## Summary

While evidence has been presented on the utility of enhancing, extending, and expanding physical activity opportunities, when improperly used or embedded within larger intervention frameworks, they can be ineffective. Hence, not all studies that incorporate one or more of these have been successful. How and where they are applied is, therefore, critical for success. Studies that expand opportunities outside of the regular school day report problems with attendance, but youth who do attend the program are more active than on non-attendance or non-program days. Approaches embedded within a compulsory environment (e.g., infusing PA into academic classrooms, enhancing required PE classes, making improvements to the playground for recess) impact all youth to increase their physical activity but issues arise with whether those in charge (e.g., school principals, after school program directors) are willing to reallocate time in a schedule to increase opportunities for youth to be active. Interventions need to be designed with these considerations in mind.

In conclusion, the TEO offers a new way to understand youth physical activity behaviors across all settings where youth physical activity is intervened upon. The TEO clarifies previously unnoted relationships in the literature with the explicit identification of fundamental mechanisms accounting for change in physical activity within youth physical activity interventions and presents a taxonomy by which to classify and identify appropriate targets for interventions designed to increase physical activity. Given the strong pragmatic nature of the theoretical components and the ease in which both intervention scientists and practitioners can incorporate them readily into existing interventions (or magnify their current standing when combined with classic inter- and intra-individual behavioral theories), we believe this formalization of the TEO will propel it to the forefront in the design of future intervention studies and, through their use, lead to a greater impact on youth activity behaviors than what has been demonstrated in previous studies. We look forward to seeing the TEO receive greater attention as the field moves forward in addressing youth physical inactivity.

## Abbreviations

MVPA: Moderate-to-vigorous physical activity
PE: Physical education
TEO: Theory of expanded, extended, and enhanced opportunities
